# Body dysmorphic disorder: A diagnostic challenge in adolescence

**DOI:** 10.4102/sajpsychiatry.v24i0.1114

**Published:** 2018-06-28

**Authors:** Yanga Thungana, Karis Moxley, Anusha Lachman

**Affiliations:** 1 Department of Psychiatry, Stellenbosch University, South Africa; 2 Department of Psychiatry, Walter Sisulu University, South Africa

## Abstract

Body dysmorphic disorder (BDD) is a chronic and disabling condition that is characterised by distressing preoccupations with perceived defects in one’s own appearance, which might be slight or not observable to others. It is considered to be an obsessive–compulsive spectrum disorder and is associated with depression, feelings of shame and poor quality of life. It is primarily a disorder of childhood or adolescent onset, and sub-clinical BDD symptoms begin, on average, several years before an individual’s symptoms meet full criteria for the disorder. Here we report the case of an adolescent admitted to an inpatient psychiatric unit for treatment of psychotic symptoms that were poorly responsive to standard treatments. This challenging case of BDD in an adolescent highlights the various comorbidities of the disorder, as well as the difficulties associated with BDD diagnosis.

## Background

Body dysmorphic disorder (BDD) is a common, chronic and disabling disorder that is often under-recognised and misdiagnosed. It consists of severe preoccupations with one or more defects in physical appearance, which are objectively slight or not noticeable to others. The concerns result in feelings of shame or unworthiness, psychological distress or depression, and poor quality of life.^1^ Body dysmorphic disorder is often diagnosed in adulthood but is primarily a disorder of childhood or adolescent onset. The average age of onset is 12–13 years.^2^ Sub-clinical BDD symptoms begin, on average, several years before individuals experience symptoms that meet the full criteria for the disorder.^3^ Early onset is associated with gradual onset, severe BDD symptoms, a lifetime history of attempted suicide and greater comorbidity which can include a lifetime eating disorder, lifetime substance use disorder, lifetime anxiety disorder and personality disorder, especially borderline personality disorder.^3^ Body dysmorphic disorder often goes undetected because many patients do not disclose their symptoms or seek help. This is mainly because of shame, lack of knowledge about BDD or a desire to avoid the problem. Furthermore, clinicians often do not identify BDD during a routine examination. This is because patients are more likely to present in a dermatological or cosmetic setting than a psychiatric one, and patients are more comfortable to reveal the comorbid symptoms, such as depression or anxiety.^3,4^

In the following report, we describe a challenging case of BDD in an adolescent female. This patient initially presented with what was thought to be a treatment-resistant primary psychotic disorder on a background of environmental adverse exposures (perinatal HIV and substance exposure, early debut of illicit substance use, early losses and sexual assault). Following the patient’s poor response to the standard antipsychotic treatment and a review of the symptomatology, we considered a diagnosis of BDD. Overall, we hope to highlight the various comorbidities and difficulties associated with BDD diagnosis.

## Case introduction report

Patient AM, a 15-year-old African female, was admitted to a psychiatric ward in a district hospital in 2014. AM was born to a teenage (15-year-old) HIV-infected mother who regularly abused illicit substances (alcohol and cannabis) during the pregnancy. AM was HIV exposed but uninfected (i.e. born HIV-negative) and was placed in the care of her maternal grandmother following her mother’s death from an HIV-related illness. At the age of 13, AM started experimenting with cannabis and then using regularly, which resulted in a history of aggression and intermittent psychotic symptoms. AM also reported a stressor of sexual assault by one of her acquaintances 3 months prior the admission.

Following admission to the psychiatric ward, AM was assessed to have a cannabis-induced psychotic disorder. Her symptoms remitted after 3 months of inpatient treatment with an atypical antipsychotic drug (risperidone), and she was discharged with a diagnosis of schizophreniform disorder. AM attended a psychosocial rehabilitation programme and was followed up on a monthly basis as an outpatient. She continued to use cannabis on discharge, which resulted in her being admitted to a drug rehabilitation programme for 6 weeks. She abstained from cannabis and continued with her antipsychotic treatment at a local community health care centre.

During a routine follow-up session in January 2016, AM presented with depressed mood, anhedonia, lack of motivation and body image complaints. She was assessed to have had a major depressive episode, with possible psychotic features. Antidepressant treatment with fluoxetine was initiated and optimised; however, she remained depressed after 3 months. She was referred for psychotherapy for persistent suicidal ideations.

In June 2016, AM reportedly stopped her treatment because she believed it made her gain weight. Despite an improvement in her depressive symptoms, she frequently missed school and felt self-conscious about her weight and breast size. AM reported being bullied at school as a result of her appearance and became preoccupied with the perceived large breasts and weight gain. She began to socially isolate herself and threatened to take an overdose of her medication. Her mood deteriorated, and she had two suicide attempts. Doctors at the local clinic stopped her oral medication and started AM on an injectable medication (flupenthixol) to minimise the risk of overdose.

By October 2016, AM had deteriorated both socially and academically. AM had stopped going to school and would not leave the house because she believed people were talking about and laughing at her large breasts. AM isolated herself and refused to interact with family members or leave the house. She remained in a dark room at home, bathed only at night and wore loose-fitting clothes to prevent family members from noticing her physical appearance. AM compared her breasts to those of her 13-year-old sister and began restricting food intake to lose weight with the hope that this would also reduce breast size. Food intake was severely restricted to only water and few fruits a day. AM was happy with the rate of weight loss she achieved, but complained that this seemed to make her breasts look even larger. Nevertheless, AM continued to lose more weight (body mass index [BMI] 25 kg/m^2^).

AM also started to complain of yellow teeth, and she began to cover her mouth when she spoke. She constantly looked in the mirror to check the colour of her eyes and teeth, with the belief that her eyes and skin had dark spots. She requested treatments involving bleaching of her skin and whitening of her teeth and sclera. She thought that she was ugly and did not want to live. Further information from family revealed that AM had displayed similar behaviour 2 years earlier. The family had observed that AM would wrap a cloth around her breasts to make them look smaller in her school uniform. Because of further deterioration in mood and escalating suicide threats, AM was involuntarily admitted to a tertiary adolescent psychiatric unit in November 2016.

## Ethical considerations

This case study was approved by the Health Research Ethics Committee of Stellenbosch University (ref no: C17/07/008) and we were granted a waiver of informed consent. We have used a code name to protect the identity of the patient. Patient anonymity and confidentiality was maintained throughout the management of this case and preparation of the report.

## Admission and assessment

AM was brought to the hospital under the guise that she was going to have breast reduction surgery. In the ward, AM refused to change clothes as she believed that the ward clothes accentuated her large breasts. She maintained a physical stance that would not draw attention to her breasts, and one hand covered her mouth at most times. Personal hygiene was not optimal because of her hesitancy to be seen undressed by other people. She appeared to spend much time preoccupied with her perceived defects, but she displayed no compulsive behaviour other than the persistent covering of her mouth. On examination, her breasts were not found to be disproportionally large or disfiguring, nor were her eyes and teeth found to be excessively discoloured. Her beliefs about her body were assessed to be exaggerated.

AM made constant attempts to isolate herself from other patients and spoke only if her hand covered her mouth. She had fixed delusional beliefs about her perceived body defects, and poor insight. She also had delusions of reference or referential thinking, and occasional inappropriate laughter, but had no thought form disorder or perceptual disturbances. During group activities, AM would sit at the back of the group to avoid being looked at. She did not participate in group activities and would complete tasks alone. During individual sessions, AM was found to be as competent as the other adolescents. When not supervised, AM continued to restrict food intake and occasionally induced vomiting. She continued to threaten suicide if she did not receive cosmetic surgery. Once it became apparent that she would not undergo surgery, AM’s mood further deteriorated and suicidal ideation increased.

On psychometric testing, AM scored 37 on the Yale-Brown Obsessive Compulsive Scale modified for Body Dysmorphic Disorder (BDD-YBOCS), which was interpreted as severe BDD. She scored 17 on the Hamilton Depression Rating Scale (HAM-D), which was interpreted as moderate depression. The Brown Assessment of Belief Scale showed that she lacked insight and was delusional.

## Treatment rationale and outcome

AM was restarted on the risperidone as a schizophreniform disorder was the original working diagnosis. She did not show a significant response, so treatment was subsequently changed to olanzapine. Despite optimisation of the antipsychotic, AM became increasingly distressed about her appearance but she remained coherent, and no formal thought disorder was identified. A selective serotonin reuptake inhibitor (SSRI) (fluoxetine) was added following the assessment of BDD with delusional beliefs without insight. Fluoxetine was well tolerated, and AM showed a noticeable reduction in her BDD symptoms. Because of poor insight, lack of cooperation and suicidality, cognitive behavioural therapy (CBT) was not feasible initially. However, CBT sessions were initiated following the improvement in AM’s condition due to pharmacotherapy. AM’s prognosis is guarded as she remains fixated on the belief that her breasts are too large and that she needs to reduce her weight. She still admits that suicide is the only option should she not undergo breast reduction surgery.

## Discussion

In the DSM-5, BDD is classified under the new category ‘obsessive-compulsive and related disorders’, along with obsessive-compulsive disorder, trichotillomania, hoarding disorder and excoriation disorder. Moreover, there is an extension of the criteria. Now, BDD also requires that at some point during the disorder, the person has performed repetitive behaviours (e.g. mirror checking, grooming and camouflaging) or mental acts (e.g. comparing appearance with that of others) in response to the perceived physical defects. A specifier indicating the degree of insight regarding BDD beliefs has been added. Therefore, the delusional variant of BDD is no longer considered a delusional disorder but rather as a specifier.^3^

Body dysmorphic disorder is often diagnosed in adulthood; however, as demonstrated in this case, BDD is primarily a disorder of childhood or adolescence onset and that sub-clinical BDD symptoms begin, on average, several years before individuals experience symptoms meeting full criteria for the disorder.^3^ The average age of onset is 12–13 years,^2^ and early onset is associated with gradual onset, severe BDD symptoms, a lifetime history of attempted suicide and greater comorbidity which can include a lifetime eating disorder, lifetime substance use disorder, lifetime anxiety disorder and personality disorder, especially borderline personality disorder.^3^ However, the psychological functioning and impairment in quality of life are comparable between early onset BDD and late onset BDD.

There is a high lifetime prevalence of major depressive disorder (MDD) in individuals with BDD, and a current episode of MDD is associated with greater morbidity in important clinical areas, including suicidality, psychological functioning and quality of life.^5^ In addition, MDD is recurrent in most individuals with melancholia, the most common subtype of depression. In most people, the onset of BDD occurs much earlier than MDD. In contrast, some studies have found a high prevalence of BDD in depressed patients, especially those with the atypical subtype, although these findings have varied considerably.^6^ Moreover, BDD is more prevalent in those with earlier onset of depression.^6^

Pharmacotherapy and psychotherapy are efficacious in treating BDD and associated comorbidity (e.g. depression and anxiety).^7,8^ Selective serotonin reuptake inhibitors, which are considered the medication of choice for BDD, often improve the symptomatology of BDD and comorbid conditions.^8^ Moreover, a continuation of treatment after acute phase might reduce the often high relapse rate associated with discontinuation of SSRI treatment. The augmentation of SSRIs with an antipsychotic in patients with delusional beliefs, delusions of reference and who are agitated or suicidal is common in clinical practice, but the literature in this regard is very limited and controversial.^8^ Augmenting an SSRI with buspirone, a 5HT1A partial agonist, is a promising alternative.^8^ Cognitive behavioural therapy is the most studied psychological intervention for BDD, with current literature suggesting that it is effective in reducing BDD symptoms and associated comorbid conditions.^7^ The effects of CBT may be sustained after treatment completion. However, this still needs further study. Additional trials that compare CBT with pharmacological therapies, as well as their combination, are warranted.

## Recommendations

Body dysmorphic disorder is a common, chronic and incapacitating disorder that is often under-recognised and poorly treated because of the diagnostic uncertainty and comorbidities that commonly accompany the diagnosis. This case highlights the diagnostic and treatment challenges in an adolescent presenting with BDD, in the context of additional environmental stressors. Although there may be variability in presentation, BDD should be considered as a differential diagnosis. This is especially important for children and adolescents who present with multiple ill-defined comorbidities (such as low mood, suicidality and anxiety), specifically in the context of fixed somatic symptoms.
